# The standardization of clinical ethics consultation and technique’s “long encirclement” of humanity: a response to Brummett and Muaygil

**DOI:** 10.1186/s13010-021-00112-y

**Published:** 2021-10-12

**Authors:** Benjamin N. Parks, Jordan Mason

**Affiliations:** 1 Mercy College of Ohio, 2221 Madison Avenue, Toledo, OH 43604 USA; 2 Albert Gnaegi Center for Health Care Ethics, Saint Louis University, 3545 Lafayette Avenue, St. Louis, MO 63104 USA

**Keywords:** Standardization, Clinical ethics consultation, Proceduralism, Phenomenology, Credentialing

## Abstract

In their recent article, Brummett and Muaygil reject Bishop et al.’s framing of the debate over standardization in clinical ethics consultation (CEC) “as one between pro-credentialing procedural and anti-credentialing phenomenological,” claiming that this framing “amounts to a false dichotomy between two extreme approaches to CEC.” Instead of accepting proceduralism and phenomenology as a binary, Brummett and Muaygil propose that these two views should be seen as the extreme ends of a spectrum upon which CEC should be done. However, as evidenced by several inconsistencies within their article, they have failed to fully appreciate the concern animating Bishop et al.’s proposal. Additionally, because of this failure, they do not seem to realize that credentialing ethicists for CEC will only create different problems in Saudi Arabia even as it possibly solves some of the current problems they identify. In this commentary, we highlight and clarify Brummet and Muaygil’s five misunderstandings of Bishop et al. This leads us to conclude that while they claim to be advocating a middle way between proceduralism and phenomenology, in fact they would like for us to standardize another proceduralism, albeit one that incorporates some of the “qualitative” values of American bioethics.

## Background

This commentary aims to clarify and correct Brummett and Muaygil’s misconceptions of Bishop et al.’s argument against the standardization of CEC.

In their recent article, Brummett and Muaygil reject Bishop et al.’s [4, 5] framing of the debate over standardization in clinical ethics consultation (CEC) “as one between pro-credentialing procedural and anti-credentialing phenomenological,” claiming that this framing “amounts to a false dichotomy between two extreme approaches to CEC” [6]. Instead of claiming proceduralism and phenomenology as a binary, Brummett and Muaygil propose that these two views should be seen as the extreme ends of a spectrum upon which CEC should be done. Thus, they claim that “CEC can be standardized and practiced in a way that navigates *between* these two extreme views” [6]. However, as evidenced by several inconsistencies within their article, they have failed to fully appreciate the concern animating Bishop et al.’s proposal. Additionally, because of this failure, they do not seem to realize that credentialing ethicists for CEC will only create different problems in Saudi Arabia even as it possibly solves some of the current problems they identify. Finally, while they claim to be advocating a middle way between proceduralism and phenomenology, in fact they would like for us to standardize another proceduralism, albeit one that incorporates some of the “qualitative” values of American bioethics. While they supposedly advocate this route as a way to find leverage against Saudi Islamist fundamentalism, we believe they are misguided. A clarification of their misunderstandings of Bishop et al. should elucidate this. We will now briefly sketch the chief concern behind Bishop et al’s proposal and much of Bishop’s solo work before highlighting the inconsistencies in Brummett and Muaygil’s paper and examining their recommendation to bring standardization and credentialing to CEC in Saudi Arabia.

### Bishop’s concern

In both Bishop et al.’s articles on CEC [4, 5] and in Bishop’s other works in bioethics identified by Brummett and Muaygil [1–3], Bishop’s main concern is the way that modern technology comes to dominate and de-humanize people. Technology here should not be understood to be particular devices, like an MRI or ventilator, but as a way of thinking and, importantly, a totalizing system that naturalizes the technological mindset such that those caught up in the system are often unaware of it, even if they feel alienated by modern technology. The technological mindset is one that only values that which is useful, efficient, and efficacious. As Brummett and Muaygil point out, Bishop draws heavily on Heidegger [4], who in “The Question Concerning Technology” identified the essence of modern technology as the “enframing” (*Gestell)* that reduces everything to a source of power to be put to use ([9], 305). Additionally, as Jacques Ellul saw, technology [*la technique*] is the domination of a concern for efficiency and as such everything must be geared toward the efficient use and progression of technology [8]. Finally, technology is inescapable because at its most basic level it is technics, skillful knowledge put to use to manipulate the world outside of us. Technics/technology came into being at the same time or even before the human, allowing for our emergence as a species. In making this point, Bernard Stiegler pointed towards evidence that before humans developed our capacity for higher cognition, we were using sophisticated tools: human hands and the abilities they granted preceded the anatomically modern human prefrontal cortex [10].

What sets modern technology apart from these older technics is its totalizing nature: its mindset or enframing. Since, as Stiegler pointed out, technology is always ahead of us, it is shaping us, and today’s technology is so far ahead of us as well as so big and powerful that the technological mindset, the techno-logic, has come to dominate all other domains of life. Everything is organized in the technological system, nothing is separated: law, science, economics, etc. are all interconnected in the service to technique. Technological systematization extends even to people, Ellul and Stiegler argue, so as to adapt humans to the ever-changing milieu, and this adaptation is accomplished by what Ellul calls “massification”, the creation of an artificial “humanity” in which humans have lost their individuality, their particularity ([8], 334). To accomplish this adaptation, the technological system deploys what Ellul calls “human techniques,” techniques that directly operate on humans in all domains of life: education, work, sport, amusement, and medicine ([8], 344-387). Chief amongst these is propaganda, which is most directly relevant to ethics because propaganda is used to exert psychic pressure on people to ensure an orthopraxy even if it fails in bringing about agreement with an orthodoxy ([8], 363-375). The technological system does not care what one thinks so long as one does what the technological system wants. Thus, Ellul can describe technology as accomplishing “a long encirclement of men [sic]” ([8], 387).

Before moving on to Brummett and Muaygil’s misunderstandings, one last thing must be said about Bishop’s argument. His argument is oriented towards the future. At present, CEC is not fully standardized and there is room for the kind of phenomenological engagement for which he advocates. However, his concern is that given the history of technology and certain current trends in CEC, such as increasing standardization and credentialing expectations, CEC will become one more technique used to ensure the orthopraxy of efficiency in health care. His concern is that moral inquiry will meet the fate described by Ellul of “sexuality, spirituality, and the capacity for feeling;” like those passions, when moral inquiry attempts to assert itself it will be “flung against a ring of iron with which technique surrounds” and become one more technique that “attacks man, impairs the sources of his vitality, and takes away his mystery” ([8], 415). It is one of the goals of the technological system to do away with mystery so as to make humans more manageable, more efficient. There are large and powerful forces at work pulling health care into ever more efficient order. And within the profession of bioethics, too, the measures of technological expediency and repeatability (“quality improvement”) are finding fertile soil.

### Brummett and Muaygil’s misunderstandings

In making their argument, Brummett and Muaygil offer five pieces of evidence that Bishop is presenting a false dichotomy and that it is possible for CEC to be standardized without collapsing into proceduralism in service to the efficient operation of health care. We believe each of these five represent misunderstandings of Bishop et al.’s argument. First, they claim that Bishop is wrong to think that credentialing will lead to ethicists quantifying every aspect of CEC even the qualitative dimensions. Here they offer evidence from ethics services in three states. The service in Illinois “has published on the aspects of their service that are amenable to quantification. Ethicists employed at the service have not come to regard what can be quantified as the only important parts of consultation practice” [6]. At first glance it seems that the ethicists are in fact in a position to avoid the proceduralist trap. However, recalling what Ellul said about propaganda above, for the efficient operation of the technological system it does not matter what the ethicists actually think so long as they act correctly. It is assumed that the efficacious, repeatable technique, when performed correctly, will yield the proper result. Bishop never suggested that ethicists would stop caring about the qualitative aspects of CEC but that proceduralism would lead to them being unable to engage in practices that will emphasize those aspects, because procedures reliably de-emphasize the qualitative. Another of Brummet and Muaygil’s examples is a service in California “where the procedural approach is markedly influential,” yet the “clinical ethicists are equally invested in enhancing the consultation experience, with all its messy human elements, for patients and practitioners alike” [6]. What at first seems to be a strong piece of evidence for their case is immediately undermined when they add that, “Through non-formal debriefing sessions, consultants attempt to better understand stakeholders’ perceptions of the consultation process in order to better optimize CEC” [6]. The ethicists at the California practice are already in the process of attempting to make the qualitative human aspects of CEC more efficient (a typical goal of techno-logic), and using the word “optimize” does not change the nature of the undertaking. Just because their discussions about optimization are “non-formal” does not mean they are any less engaged in the prevailing logic of technique. “Optimization” implies a “best” way of doing CEC, and once one has found the best way it will be standardized and quantified, even if it was discovered through non-formal dialogue.

Two, Brummett and Muaygil point to some ethicists’ suggestion that future iterations of the HEC-C certification exam should include “ways to evaluate a practitioner’s *qualitative skills* such as open-ended questions, a portfolio demonstrating more experience, or completion of a formal consultation program” as evidence that “ethicists do not seem tempted to confuse what can be easily quantified with the qualitative aspects that are required to do consultation well” [6]. This indicates nothing of the sort and may even prove Bishop’s points. Open-ended questions on an exam must be quantified in order to assign a grade. As for experience, how will it be deemed adequate for certification? Most likely through a set number of hours. Any “formal consultation program” will need to be one that is in line with those responsible for credentialing ethicists – as is the case with any program preparing people for certification in any other field. If the proceduralist mindset is what is driving certification, acceptable programs for certification will be proceduralist. It is the nature of tests and certification procedures to quantify – to encircle the human – otherwise assigning a grade is impossible. However, debates about what should go on the certification exam ultimately do not matter for CEC because ethicists do not run the hospitals and are constantly influenced by the organs of the technological system. Ultimately, the business, legal, and medical parts of the hospital will integrate CEC into a more efficiently run hospital, and certification is a means to that end.

Three, Brummett and Muaygil gesture towards the Core Competencies Report’s endorsement of the facilitation approach which “calls for mediating the resolution of moral dilemmas *within the range of ethically acceptable options*” [6]. However, we may ask what is exactly considered to be “within the range of ethically acceptable options.” There can be more than one outcome that is sufficiently efficient for a health system. Thus, having a range of options is not enough to ease Bishop’s concern that those options will be delimited by technocratic interests. There is another problem with the facilitation approach that we will simply mention here: the method itself strongly resembles Maoist propaganda techniques that ensure compliance with the propagandist’s direction while convincing the followers that they themselves have chosen what to believe for themselves ([7], 308-309 cf. 79-84). That is to say, facilitation is not itself free of morally-weighted technique.

Four, Brummett and Muaygil think that Bishop contradicts himself by describing a method for doing CEC after criticizing standardization. However, Bishop never says that ethics should be free of technique. He would wholeheartedly agree with Brummett and Muaygil’s assertion that “process and content need not be thought of as binary approaches to CEC.”

What matters, though, is the kind of technique employed in CEC. It is precisely because Bishop believes process and content are intertwined that he is so concerned about the tyranny of technique in bioethics; as we engage in techniques and enter into their enframing, our moral horizons are shaped by them and the content of our ethics is brought into form. Bishop is deliberately suggesting a technique that resists, rather than assumes, rote proceduralism in service to efficiency. In seeking such a technique, Bishop is in line with even the greatest critics of technology in realizing that technique is unavoidable but can be done in a more or less human way. Even Heidegger wrote about two types of techne: *poesis* (the technique Bishop advocates) and challenging-forth (the technique he critiques). By yelling *tu quoque*, Brummett and Muaygil have accomplished nothing other than to point out something that a long line of philosophers has already recognized.

Finally, they suggest that credentialling is important for overcoming legalistic (religious legalism specifically) and exclusivist (namely patriarchal and paternalistic) approaches to bioethics that negatively affect the wellbeing of those that occupy a lower place in society. Here they pivot to the current state of bioethics in Saudi Arabia, which they claim “illustrate [s] the importance of standardizing the practice of CEC” [6]. After recounting the shortcomings of Saudi bioethics, they claim that “Saudi bioethics could be improved through standardization and that applying the phenomenological approach here, with its decidedly anti-credentialing stance and vague insistence on what is ‘local and particular’ to a case, would only serve to maintain the status quo” [6]. Yet again, they show that they have not fully appreciated Bishop’s arguments. Saudi bioethics, with its Islamic fundamentalist bent, is not concerned with the local and the particular. Such is the nature of any religious fundamentalism whether it is Islamic, Christian, Jewish, etc. The individual person and his or her circumstances are effaced and the rigid interpretation of scripture or some other teaching are applied. Bishop’s critique of CEC credentialing and proceduralism would apply equally to the current state of Saudi bioethics.

In fact, Brummett and Muaygil concede that very point when they write, “We contend that Bishop et al’s concerns of an unreflective practice, uninformed process, and unfulfilled potential – as levied against the procedural view – are not far from the current realities of Saudi Bioethics” [6]. However, instead of exploring how Bishop’s proposed solution to proceduralism could benefit Saudi bioethics they claim in the next sentence that “Credentialing, in this context, could be a solution to these problems, not the cause of them” [6]. It is hard to see how that would be the case; through an act of bioethical colonialism they will replace one individual-effacing proceduralism with another. While Brummet and Muaygil write of Saudi bioethics:

The focus on legal and Islamic considerations gave the DNR guidelines essential legal and religious validity. The pragmatic approach to ethical issues at the of end of life permitted clinicians a certain comfortable practicality. Still, by allowing bioethics only a nominal presence, fundamental moral claims about life and death were neglected [6].

This could just as easily be restated as:

The focus on legal and procedural considerations gave the ASBH guidelines essential validity. The pragmatic approach to solving ethical issues in medicine permitted ethicists and clinicians a certain comfortable practicality. Still, by allowing robust bioethical debate and phenomenological encounter only a nominal presence, fundamental moral claims about life and death were neglected.

Perhaps Brummet and Muaygil do not disagree with Bishop et al. as much as they think.

## Conclusion

If the above is unconvincing, consider this. Education is something highly qualitative. Yet, over the past couple decades we have seen ever increasing amounts of quantification and standardization even at the highest levels and even at schools that had been committed to non-technical education. As evidenced by faculty and program cuts, the humanities are only deemed necessary insofar as they serve STEM fields, which are in turn dominated by technique. In the medical context, bioethics occupies an analogous position to the humanities. It is Bishop’s contention that the standardization of bioethics will strengthen the grip of technique on bioethics, because while moral mastery cannot be measured, action can be evaluated. Bioethics is always already in service to technique, and the question is how much freedom it can carve out for moral inquiry that is not in service to the efficient operation of a health system. While they claim to be advocating a middle way between proceduralism and phenomenology, in fact Brummett and Muaygil would like for us to standardize another proceduralism. Ultimately, they misunderstand Bishop’s claims and overestimate how much freedom bioethics has.

## Data Availability

Not applicable.

## Abbreviations

CEC: Clinical Ethics Consultation
ASBH: American Society for Bioethics and Humanities
DNR: Do Not Resuscitate
